# Neutral Impact of SARS-CoV-2 Coinfection on the Recombination-Driven Evolution of Endemic HCoV-OC43

**DOI:** 10.3390/v17091263

**Published:** 2025-09-18

**Authors:** Xueling Zheng, Yinyan Zhou, Yue Yu, Shi Cheng, Feifei Cao, Zhou Sun, Jun Li, Xinfen Yu

**Affiliations:** 1 Hangzhou Center for Disease Control and Prevention (Hangzhou Health Supervision Institution), Hangzhou 310002, China; 2 Zhejiang Key Laboratory of Multi-Omics in Infection and Immunity, Hangzhou 310002, China

**Keywords:** co-infection, SARS-CoV-2, HCoV-OC43, HCoV-HKU1, evolutionary recombination, emergent lineage

## Abstract

Knowledge gaps exist on whether SARS-CoV-2 co-infection alters recombination frequency or induces phylogenetic incongruities in endemic β-coronaviruses (HCoV-OC43, HCoV-HKU1), limiting our understanding of cross-species evolution. Among 7213 COVID-19 and 1590 non-COVID-19 acute respiratory cases (2021–2022) screened via multiplex PCR, β-coronavirus co-infections (SARS-CoV-2 + HCoV-OC43/HKU1) and single HCoV-OC43/HKU1 infections were identified. Whole-genome sequencing (Illumina NovaSeq) was performed. Phylogenies were reconstructed using Bayesian inference (MrBayes). Recombination was assessed via Bootscan analysis (SimPlot). Co-infection prevalence was low (0.51%, mainly HCoV-HKU1: 0.28%, HCoV-OC43: 0.11%). HCoV-OC43 diverged into lineage 1 (genotype K) and a novel recombinant lineage 2 (genotypes F/J/G/I segments), exhibiting accelerated evolution. HCoV-HKU1 remained genetically stable (genotype B). Co-infection status did not influence evolutionary outcomes. While SARS-CoV-2 co-infection may favor transmission of endemic HCoVs, their evolution appears driven by population-level selection, not co-infection. HCoV-OC43 underwent recombination-driven diversification, contrasting sharply with HCoV-HKU1’s stasis, highlighting distinct evolutionary strategies. Integrated genomic and clinical surveillance is critical for tracking coronavirus adaptation.

## 1. Introduction

Human coronaviruses (HCoVs) represent a diverse group of viruses capable of inducing respiratory illnesses ranging from mild common colds to severe respiratory syndromes. Currently, seven distinct HCoVs have been characterized, classified into two α-coronaviruses (α-CoVs) and five β-coronaviruses (β-CoVs) [1]. These viruses demonstrate remarkable adaptive potential through frequent spike protein mutations, facilitating cross-species transmission and enhancing infectivity [2]. Notably, the β-coronavirus SARS-CoV-2 emerged as a global health crisis, causing the COVID-19 pandemic that has resulted in over 777 million confirmed cases and 7.09 million fatalities worldwide as of April 2025 (https://covid19.who.int/ accessed on 12 April 2025 ).

Pathogen co-infection could present significant clinical challenges, including immune system compromise, therapeutic efficacy reduction, and disease prognosis deterioration [3]. Co-infection scenarios create optimal conditions for viral recombination during replication, particularly through template-switching mechanisms that generate chimeric sub-genomic RNAs [2]. The SARS-CoV-2 pandemic has already demonstrated this phenomenon through recombinant lineages XD (Delta-Omicron hybrid) and XE (BA.1-BA.2 recombinant), which fuelled successive infection waves [4,5]. While numerous studies have documented co-infections involving SARS-CoV-2 and other pathogens [6,7,8,9,10], limited data exist regarding co-infection with endemic β-coronaviruses (HCoV-OC43 and HCoV-HKU1). These common HCoVs typically cause self-limiting upper respiratory tract infections but can progress to moderate-severe disease in vulnerable populations (infants, elderly, and immunocompromised individuals) [11,12], accounting for 15–30% of adult common cold cases [13].

This study investigates the evolutionary implications of their co-infection between SARS-CoV-2 and β-coronaviruses (HCoV-OC43 and HCoV-HKU1). Through comparative analysis of co-infected versus mono-infected β-coronavirus cases (non-SARS-CoV-2 positive controls), we aim to elucidate potential recombination events and evolutionary pressures arising from viral co-circulation. Our findings will inform precision therapeutic strategies, enhance understanding of seasonal transmission patterns, and establish a framework for molecular surveillance of coronavirus evolution. This research addresses critical gaps in our knowledge of coronavirus interactions, particularly relevant in the context of persistent SARS-CoV-2 circulation alongside endemic HCoVs.

## 2. Materials and Methods

### 2.1. Patient Characteristics

This retrospective cohort study analyzed 7213 RT-PCR-confirmed SARS-CoV-2 positive nasopharyngeal swabs collected from patients at the Hangzhou Center for Disease Control and Prevention between 2021 and 2022. The cohort exhibited distinct demographic stratification, with female patients accounting for 36.1% (2604) and males comprising 63.9% (4609) of the samples. A striking temporal disparity was observed, with 2022 specimens outnumbering 2021 cases by nearly tenfold (ratio 1:9.85). Age distribution analysis revealed a predominance of young adults, with 67.68% of cases occurring in individuals aged 18–44 years, followed by 21.36% in the middle-aged population (45–60 years). Pediatric (<18 years: 5.64%) and elderly (>60 years: 5.31%) populations constituted substantially smaller proportions of the cohort. In addition, a total of 1590 non-COVID-19 acute respiratory cases were included as controls without co-infection with HCoV-OC43 and HCoV-HKU1, among which 40 cases were positive for HCoV-OC43 and 13 for HCoV-HKU1.

### 2.2. Nucleic Acid Extraction and Human Coronavirus Molecular Detection

Viral RNA extraction and detection were performed using standardized laboratory protocols. Briefly, RNA was isolated from 200 µL of clinical specimens using the TianLong RNA Extraction Kit (TianLong Science and Technology Co., Xi’an, China), followed by nucleic acid amplification and detection on the Applied Biosystems QuantStudio™ 5 Real-Time PCR System (Thermo Fisher Scientific, Waltham, MA, USA). The subsequent analysis employed a multiplex RT-PCR assay (Quadruplex Coronavirus Detection Kit, XABT Biotechnology, Beijing, China) for simultaneous identification of four human coronaviruses: HKU1, OC43, 229E, and NL63. All experimental procedures were conducted in strict accordance with the manufacturers’ standardized protocols.

### 2.3. Complete Genome Sequencing and Genome Analysis

Successful whole genome sequencing was achieved for 6 HCoV-OC43 and 8 HCoV-HKU1 strains from co-infected individuals, with additional 8 HCoV-OC43 and 3 HCoV-HKU1 genomes obtained from mono-infected cases. Sample exclusions were attributed to suboptimal nucleic acid concentrations or quality degradation. Specifically, the sequencing workflow comprised three principal phases: (1) Target enrichment: human coronavirus genomes were amplified using a multiplex PCR amplification system (BioGerm Medical Technology, Shanghai, China); (2) Library preparation: amplified products underwent purification (QIAquick PCR Purification Kit, Qiagen, Hilden, Germany), fragmentation to ~200 bp (Covaris M220), and library construction (Nextera XT DNA Library Prep Kit, Illumina, San Diego, CA, USA); (3) Sequencing execution: sequencing was performed using Illumina NovaSeq (PE150 mode) ( Illumina, San Diego, CA, USA) for HCoV-OC43 and HCoV-HKU1, following established protocols. Raw sequencing data underwent quality assessment using FastQC (v0.11.9) with default parameters. Sequence assemble employed Bowtie2 (v2.7.2) against HCoV-OC43 (NC_006213 references) and HCoV-HKU1 (NC_006577 references). Post-alignment processing included format conversion (SAMtools v1.15.1) and variant calling using BCFtools (v1.15.1) with stringent thresholds (Phred quality ≥ 20, depth ≥ 20×).

### 2.4. Genetic Characterisation and Phylogenetic Analysis

To elucidate the evolutionary relationships between β-coronaviruses (β-CoVs) identified in our study and global strains, we conducted comprehensive phylogenetic analyses of complete genome sequences for both HCoV-OC43 and HCoV-HKU1. For HCoV-OC43, our obtained sequences were aligned with reference sequences representing genotypes A-K, while HCoV-HKU1 sequences were compared against genotypes A–C references (Supplementary Tables S1 and S2), using the MAFFT algorithm (v7.520) implemented in Geneious Prime (2024.0.4) with default parameters. Bayesian inference was performed using MrBayes v3.2.7, employing the General Time Reversible (GTR) substitution model with gamma-distributed rate variation (+G) and a proportion of invariant sites (+I), as determined by hierarchical likelihood ratio tests in MrModelTest v2.4. To quantify genetic divergence between genotypes, we calculated pairwise genetic distances using the p-distance model with 1000 bootstrap replicates in MEGA v11.0. This analysis incorporated complete genome sequences from all available inter-genotype comparisons, ensuring comprehensive assessment of sequence heterogeneity.

### 2.5. Recombination Analysis

To investigate potential recombination events and precisely map breakpoint locations across HCoV-OC43 and HCoV-HKU1 genomes, we conducted Bootscan analysis using SimPlot v3.5.1 with the following optimized parameters: Kimura 2-parameter substitution model, 1000 bootstrap replicates, 1000 bp sliding window, and 200 bp step increment. For subsequent phylogenetic validation of recombination signals, we implemented a partitioned Bayesian analysis strategy for HCoV-OC43. The genome was segmented into six distinct sub-regions (I–VI) based on identified recombination breakpoints. Evolutionary models were independently selected for each partition through hierarchical likelihood ratio tests: (1) Sub-regions I, II, III, and V: GTR with invariant sites (GTR + I); (2) Sub-region IV: GTR without rate heterogeneity parameters; (3) Sub-region VI: GTR with invariant sites and gamma-distributed rates (GTR + I + G).

## 3. Results

### 3.1. Case Characteristics

Our analysis identified 37 co-infection cases among 7213 specimens, yielding an overall co-infection prevalence of 0.51% (37/7213). Stratification by coronavirus type revealed distinct co-infection frequencies: HCoV-HKU1 demonstrated the highest rate at 0.28% (20/7213), followed sequentially by HCoV-OC43 (0.11%, 8/7213), HCoV-229E (0.10%, 7/7213), and HCoV-NL63 (0.03%, 2/7213) (Table 1). Demographic analysis showed significant association between male sex and increased HCoV detection rates, particularly for co-infected cases involving HCoV-OC43 and HCoV-229E (Table 1). The SARS-CoV-2 positive cohort exhibited a mean age of 37.56 years. Age distributions varied among co-infected subgroups: 35.15 ± 14.02 years for HCoV-HKU1, 20.88 ± 13.09 years for HCoV-OC43, 34.14 ± 15.75 years for HCoV-229E, and 26.00 ± 2.83 years for HCoV-NL63. Notably, non-SARS-CoV-2 surveillance data demonstrated temporal variations in prevalence: 0.7% in 2021 vs. 0.8% in 2022 for HCoV-HKU1, and 2.4% in 2021 vs. 2.6% in 2022 for HCoV-OC43.

### 3.2. Phylogenetic Analysis

**Figure 1:**
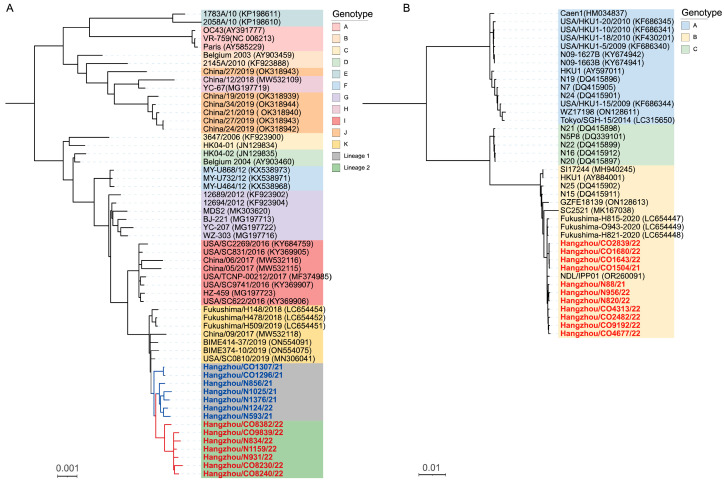
Bayesian phylogenetic reconstructions of complete genome sequences for (**A**) HCoV-OC43 and (**B**) HCoV-HKU1. (**A**) Terminal taxa are annotated with lineage-specific identifiers—blue bold denotes lineage 1 (dominant 2021 clade) and red bold designates lineage 2 (emerging 2022 variant). (**B**) Highlighted red branches represent study strains from Hangzhou, China. Strain identifiers incorporate co-infection status (CO: co-infected; N: non-coinfected). The scale bar indicates nucleotide substitutions per site (subs/site).

Our phylogenetic investigation incorporated complete genome sequences of global reference strains for HCoV-OC43 (genotypes A–K) and HCoV-HKU1 (genotypes A–C) retrieved from GenBank (Supplementary Tables S1 and S2). The HCoV-OC43 phylogeny revealed an even distribution between lineage 1 (50%) and lineage 2 (50%), both descending from a genotype K-like ancestor (bootstrap support 100%) (Figure 1A). Inter-genotype distance analysis demonstrated substantial divergence (>0.7% [0.007 subs/site]) between lineages 1/2 and genotypes A–B/C/D/E/H/J, contrasted with closer relationships (<0.5% [0.005 subs/site]) to genotypes F/G/I/K (Table 2). Notably, the genetic distances between genotype K and lineages 1 (0.13%) and 2 (0.18%) were found to be below the established 0.29% threshold for distinguishing HCoV-OC43 genotypes [14], defined as the minimal inter-genotype divergence reported in that study. This strongly supports the hypothesis that lineages 1 and 2 represent recent evolutionary derivatives of genotype K. Phylogenetic analysis of HCoV-HKU1 revealed three well-supported genotype clusters (A–C) with substantial nucleotide divergence: 5.01% between genotypes A and B, 3.85% between A and C, and 2.01% between B and C. Notably, all Hangzhou strains demonstrated minimal genetic divergence from genotype B (0.3% [0.003 substitutions/site]), confirming their classification within this genotype (Figure 1B).

**Figure 2:**
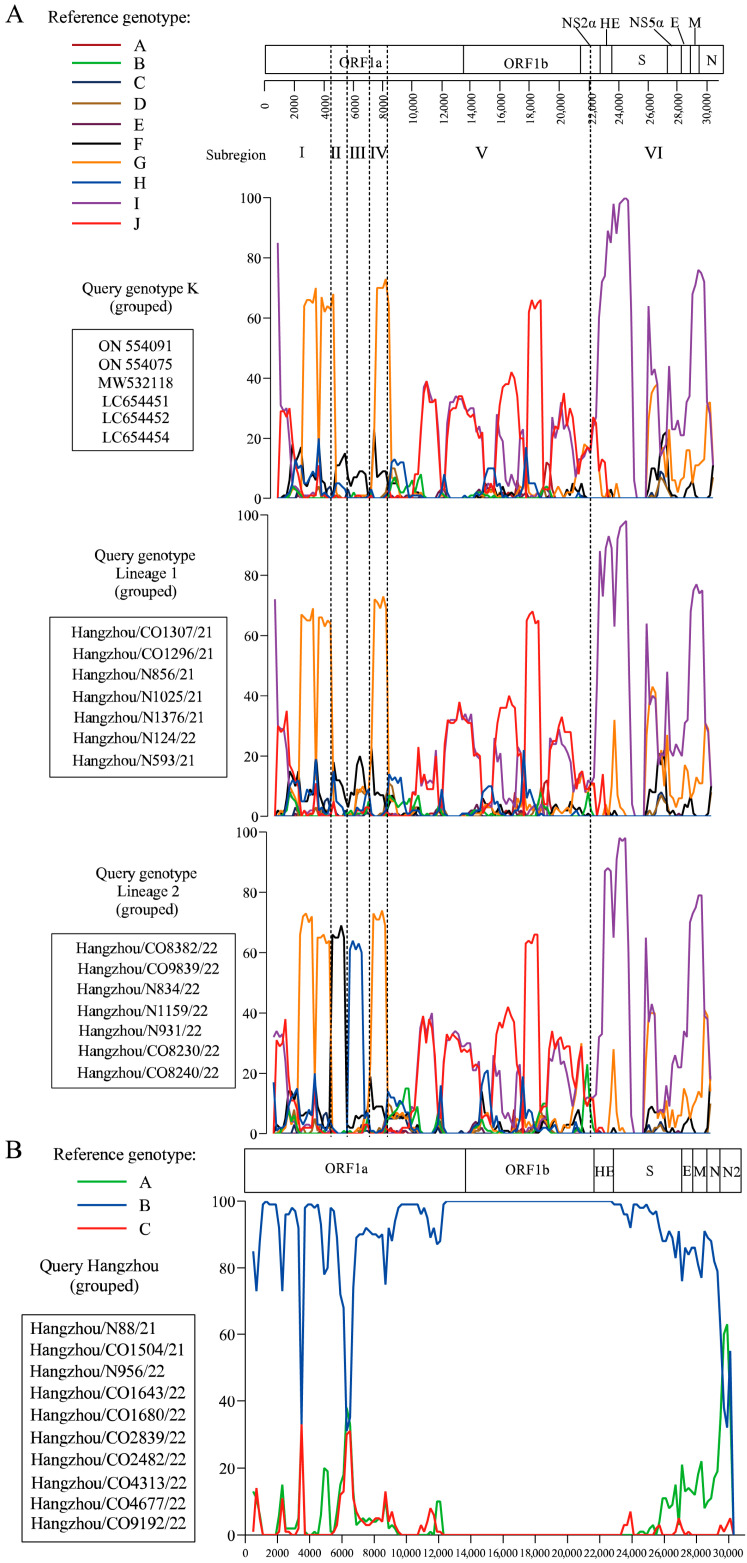
Genomic recombination analysis of HCoV-OC43 and HCoV-HKU1. (**A**) Bootscan analysis of HCoV-OC43 genomes comparing recombination patterns between genotype K, lineage 1, and lineage 2. Reference genomes from genotypes A–J were used as putative parental strains. (**B**) Bootscan analysis of HCoV-HKU1 complete genome was performed using reference genomes for genotypes A, B and C as putative parental genotype. Co-infection status (CO: co-infected; N: non-coinfected).

In addition, our analysis revealed three critical insights into co-infection dynamics and viral evolution: (1) Co-infected and mono-infected strains formed overlapping clades, demonstrating no significant phylogenetic differentiation between groups. This suggests SARS-CoV-2 co-infection does not exert detectable evolutionary pressure on HCoV genomic evolution (Figure 1A,B); (2) Lineage 1 maintained established clade dominance in 2021 samples (excluding Hangzhou/N124/22), and Lineage 2 emerged as the predominant variant in 2022, which suggested that seasonal lineage replacement accompanies measurable evolutionary acceleration (Figure 1A); (3) Lineage 2 demonstrated significantly greater genetic divergence from historical genotypes compared to lineage 1, indicative of accelerated evolutionary rates and emergent variants can rapidly establish dominance during the 2021–2022 surveillance period (Table 2).

### 3.3. Recombination Analysis

Bootscan analysis of HCoV-OC43 lineages 1 and 2 revealed distinct recombination patterns when compared to genotype K, using full-length reference genomes from genotypes A–J as potential parental strains (Figure 2A and Figure S1). Lineage 1 exhibited five genomic subregions with clear phylogenetic affiliations: subregions I (1–4300 nt) and IV (6500–8100 nt) aligned with genotype G, subregion V (8100–22,300 nt) with genotypes J/I, and subregion VI (22,300–3′ end) with genotype I. In contrast, lineage 2 displayed increased recombination complexity, featuring six subregions with an additional breakpoint in subregion II (4300–5500 nt) and subregion III (5500–6500 nt) linked to genotype F and H, respectively. This finding suggests progressive recombination complexity during the 2021–2022 evolutionary transition. Comparative analysis of co-infected (CO) and non-coinfected (N) strains demonstrated no structural divergence in mosaic recombination patterns for either HCoV-OC43 or HCoV-HKU1 (Figure 2A,B). Notably, HCoV-HKU1 strains exhibited genomic stability, with Bootscan analysis confirming the absence of detectable recombination events and >99.7% sequence identity to genotype B, consistent with phylogenetic clustering patterns (Figure 2B).

## 4. Discussion

The global emergence of SARS-CoV-2 has intensified scrutiny of respiratory pathogen co-infections, with numerous studies documenting its coexistence with bacterial and viral agents [9,15,16,17]. However, the interactions between SARS-CoV-2 and endemic human coronaviruses (HCoVs)—particularly β-coronaviruses such as HCoV-HKU1 and HCoV-OC43—remain poorly understood. Although co-infection with SARS-CoV-2 and HCoVs has been hypothesized to drive β-coronavirus evolution through recombination, key questions persist regarding the differential evolutionary trajectories in co-infected versus mono-infected populations. To address these gaps, this study aims to elucidate the evolutionary consequences of SARS-CoV-2 and endemic β-coronavirus (HCoV-OC43/HKU1) co-infection, with a specific focus on recombination-mediated genomic divergence compared to mono-infection scenarios. Specifically, we seek to determine whether co-infection accelerates the genomic evolution of β-coronaviruses and how these evolutionary dynamics differ between co-infected and single-pathogen infections.

The co-existence of pathogens in a single host may generate synergistic, competitive, and inhibitory virological interactions, thereby shaping distinct infection patterns [18,19,20]. In our cohort of 7213 clinical specimens, 37 cases (0.51%) exhibited co-infection events, with HCoV-HKU1 (0.28%) and HCoV-OC43 (0.11%) exhibiting the highest co-infection frequencies (Table 1). This preferential coexistence could stem from competitive ecological advantages in SARS-CoV-2-infected hosts. However, absent vaccination records and clinical outcomes preclude causal inference between co-infection status and disease progression, as well as reliable severity comparisons between co-infected and mono-infected cohorts. A striking male predominance was observed in HCoV-positive cases, consistent with prior reports linking male sex to increased coronavirus susceptibility and severe outcomes [21,22]. Interestingly, this sex disparity was more pronounced in HCoV-OC43 and HCoV-229E co-infections, hinting at potential sex-dimorphic mechanisms—whether immunological or behavioral—that may modulate co-infection risks. The clinical relevance of this observation remains ambiguous, particularly given our inability to account for vaccination status. These methodological constraints, potential confounding effects arising from the inclusion of imported cases (which may influence beta-coronavirus co-infection rates), and health-seeking behaviors, necessitate circumspect interpretation of the reported associations.

Phylogenetic analysis revealed distinct evolutionary patterns among HCoVs. HCoV-OC43, the most genetically plastic of the endemic coronaviruses, has diversified into 11 genotypes (A–K) over the past decade [14,23,24,25,26,27,28,29]. Our data delineate two temporally stratified lineages: Lineage 1 dominated 2021 samples, while Lineage 2 emerged as the predominant variant in 2022, exhibiting significantly greater genetic divergence from historical genotypes (Table 2, Figure 1A). Bootscan analysis further revealed escalating recombination complexity in Lineage 2, characterized by an additional genotype F and H -associated breakpoint in subregion II (4300–5500 nt) and subregion III (5500–6500 nt) (Figure 2A and Figure S1). This progressive genomic restructuring suggests continuous adaptation to human hosts, potentially driven by immune selection pressure. In contrast, HCoV-HKU1 demonstrated remarkable evolutionary stasis, with all study strains clustering within genotype B (99.7% identity) and showing no evidence of recombination (Figure 1B and Figure 2B). This conservation aligns with its classification into stable genotypes (A–C) [30], underscoring fundamental differences in evolutionary mechanisms among β-coronaviruses. Notably, co-infection status showed no measurable impact on recombination patterns or evolutionary trajectories for either HCoV-OC43 or HCoV-HKU1, suggesting viral evolution is primarily driven by host population-level selection rather than individual co-infection events.

Our findings should be interpreted in light of three key limitations: geographic restriction to Hangzhou populations and confounding effects of pandemic containment measures. These limitations underscore the need for expanded data collection across diverse populations. Furthermore, the absence of clinical metadata precluded severity assessments and transmission analyses of co-infected β-coronavirus strains. Future investigations should prioritize integrating virological data with standardized clinical records to resolve genotype-phenotype correlations in coronavirus infections.

## 5. Conclusions

Co-infection analysis revealed HCoV-HKU1 (0.28%) and HCoV-OC43 (0.11%) exhibit competitive dominance in SARS-CoV-2-positive hosts, with demographic analysis identifying male sex as an independent risk factor. Phylogenomic reconstruction delineated two emerging HCoV-OC43 lineages: lineage 1 (genotype K) and lineage 2, a recombinant derivative originating from genotype K through sequential integration of genomic segments from genotypes F, J, G, and I. In contrast, HCoV-HKU1 maintained genomic stability as genotype B without detectable recombination signals. Crucially, co-infection status showed no measurable impact on β-coronavirus evolutionary trajectories.

These evolutionary insights highlight the adaptive potential of HCoV-OC43 through recombination-driven diversification, while underscoring the need for surveillance systems integrating host immunological data to assess clinical impacts of emerging lineages. The conserved nature of HCoV-HKU1 suggests distinct evolutionary constraints among human coronaviruses, warranting mechanistic studies on host–pathogen coevolution.

## Figures and Tables

**Table 1:** Co-infection characteristics of HCoV-HKU1, HCoV-OC43, HCoV-229E, and HCoV-NL63 with SARS-CoV-2 in Hangzhou, China, 2021–2022.

	HCoV-HKU1	HCoV-OC43	HCoV-229E	HCoV-NL63
Male	13	8	7	1
Female	7	0	0	1
Total	20	8	7	2
Age ± SD	35.15 ± 14.02	20.88 ± 13.09	34.14 ± 15.75	26.00 ± 2.83
Co-infection	0.28%	0.11%	0.10%	0.03%

**Table 2:** Estimation of pairwise genetic distances among HCoV-OC43 and HCoV-HKU1 genotypes based on complete genome.

HCoV		Genetic Distance (%)												
OC43		A	B	C	D	E	F	G	H	I	J	K	Lineage 1	Lineage 2
	A	-												
	B	0.92	-											
	C	0.90	0.74	-										
	D	0.92	0.64	0.28	-									
	E	1.05	0.84	1.05	0.84	-								
	F	1.04	0.76	0.44	0.76	0.47	-							
	G	1.08	0.81	0.50	0.81	0.35	0.28	-						
	H	1.09	0.56	0.85	0.41	1.29	0.76	0.80	-					
	I	1.16	0.89	0.59	0.33	1.20	0.37	0.27	0.86	-				
	J	1.09	0.55	0.44	0.27	1.16	0.73	0.74	0.47	0.66	-			
	K	1.18	0.88	0.28	0.88	1.03	0.44	0.32	0.87	0.27	0.71	-		
	Lineage 1	1.21	0.92	0.75	0.92	1.32	0.47	0.35	0.90	0.31	0.75	0.13	-	
	Lineage 2	1.26	0.97	0.79	0.97	1.37	0.52	0.40	0.95	0.36	0.79	0.18	0.15	-
HKU1		A	B	C	Hangzhou									
	A	-												
	B	5.01	-											
	C	3.85	2.01	-										
	Hangzhou	5.06	0.30	2.06	-									

Note: Pairwise genetic distances were expressed by percentage (%) difference.

## Data Availability

All sequences generated in this study have been lodged in GenBank and the accession numbers are shown in Supplementary Tables S1 and S2. The raw data supporting the conclusions of this article will be made available by the authors, without undue reservation.

## Supplementary Materials

The following supporting information can be downloaded at: https://www.mdpi.com/article/10.3390/v17091263/s1, Figure S1: Putative parental genotype determination in sub-genomic regions (sub-regions I–VI) of genotypes K, lineage 1 and lineage 2 recombinants; Table S1: Background information for published and genotyped global full-length genome of HCoV-HKU1 strains used for phylogenetic analysis.; Table S2: Background information for published and genotyped global full-length genome of HCoV-OC43 strains used for phylogenetic analysis.
