# Supplementary material for: Neutral Impact of SARS-CoV-2 Coinfection on the Recombination-Driven Evolution of Endemic HCoV-OC43
Source: Viruses. 2025 Sep 18;17(9):1263. doi: 10.3390/v17091263 (PMC12474069; doi:10.3390/v17091263)
Supplement: Supplementary file 1 [file viruses-17-01263-s001.zip › viruses-3813539-supplementary.pdf]

Supplementary Materials

Neutral Impact of SARS-CoV-2 Coinfection on the Recombination-Driven Evolution of Endemic HCoV-OC43

Table S1. Background information for published and genotyped global full-length genome of HCoV-HKU1 strains used for phylogenetic analysis.

Strain name	Accession NO.	Year	Sampling sites	Genome	Genotype	Reference
Hangzhou/CO1504/21	PQ522255	2021	Hangzhou, China	Complete Genome	B	This study
Hangzhou/CO1643/22	PQ522254	2022	Hangzhou, China	Complete Genome	B	This study
Hangzhou/CO1680/22	PQ522253	2022	Hangzhou, China	Complete Genome	B	This study
Hangzhou/CO2482/22	PQ522260	2022	Hangzhou, China	Complete Genome	B	This study
Hangzhou/CO2839/22	PQ522252	2022	Hangzhou, China	Complete Genome	B	This study
Hangzhou/CO4313/22	PQ522259	2022	Hangzhou, China	Complete Genome	B	This study
Hangzhou/CO4677/22	PQ522262	2022	Hangzhou, China	Complete Genome	B	This study
Hangzhou/CO9192/22	PQ522261	2022	Hangzhou, China	Complete Genome	B	This study
Hangzhou/N88/21	PQ522256	2021	Hangzhou, China	Complete Genome	B	This study
Hangzhou/N956/22	PQ522257	2022	Hangzhou, China	Complete Genome	B	This study
Hangzhou/N820/22	PQ522258	2022	Hangzhou, China	Complete Genome	B	This study
Caen1	HM034837	2005	Caen, France	Complete Genome	A	Genbank
USA/HKU1-20/2010	KF686345	2010	Denver, USA	Complete Genome	A	Genbank
USA/HKU1-10/2010	KF686341	2010	Denver, USA	Complete Genome	A	Genbank
USA/HKU1-18/2010	KF430201	2010	Denver, USA	Complete Genome	A	Genbank
USA/HKU1-5/2009	KF686340	2009	Denver, USA	Complete Genome	A	Genbank
N09-1627B	KY674942	2016	Seattle, USA	Complete Genome	A	Genbank
N09-1663B	KY674941	2016	Seattle, USA	Complete Genome	A	Genbank
HKU1	AY597011	2004	Hongkong, China	Complete Genome	A	Genbank
N19	DQ415896	2004	Hongkong, China	Complete Genome	A	Genbank
N7	DQ415905	2004	Hongkong, China	Complete Genome	A	Genbank
N24	DQ415901	2005	Hongkong, China	Complete Genome	A	Genbank
USA/HKU1-15/2009	KF686344	2009	Denver, USA	Complete Genome	A	Genbank
WZ17198	ON128611	2018	Wenzhou, China	Complete Genome	A	Genbank
Tokyo/SGH-15/2014	LC315650	2014	Tokyo, Japan	Complete Genome	A	Genbank
SI17244	MH940245	2017	Ratchaburi, Thailand	Complete Genome	B	Genbank
HKU1	AY884001	2003	Hongkong, China	Complete Genome	B	Genbank
N25	DQ415902	2005	Hongkong, China	Complete Genome	B	Genbank
N15	DQ415911	2004	Hongkong, China	Complete Genome	B	Genbank
GZFE18139	ON128613	2018	Guangzhou, China	Complete Genome	B	Genbank
SC2521	MK167038	2017	Washington, USA	Complete Genome	B	Genbank
Fukushima-H815-2020	LC654447	2020	Fukushima, Japan	Complete Genome	B	Genbank
Fukushima-O943-2020	LC654449	2020	Fukushima, Japan	Complete Genome	B	Genbank
Fukushima-H821-2020	LC654448	2020	Fukushima, Japan	Complete Genome	B	Genbank
NDL/IPP01	OR260091	2022	Amsterdam, Netherlands	Complete Genome	C	Genbank
N21	DQ415898	2004	Hongkong, China	Complete Genome	C	Genbank
N5P8	DQ339101	2004	Hong Kong, China	Complete Genome	C	Genbank
N22	DQ415899	2005	Hongkong, China	Complete Genome	C	Genbank

N16	DQ415912	2004	Hongkong, China	Complete Genome	C	Genbank
N20	DQ415897	2004	Hongkong, China	Complete Genome	C	Genbank

Table S2. Background information for published and genotyped global full-length genome of HCoV-OC43 strains used for phylogenetic analysis.

Strain name	Accession NO.	Sampling year	Sampling sites	Genome	Genotype	Reference
Hangzhou/CO1296/21	PQ505906	2021	Hangzhou, China	Complete Genome	lineage 1	This study
Hangzhou/CO1307/21	PQ505905	2021	Hangzhou, China	Complete Genome	lineage 1	This study
Hangzhou/CO8230/22	PQ505898	2022	Hangzhou, China	Complete Genome	lineage 2	This study
Hangzhou/CO8240/22	PQ505893	2022	Hangzhou, China	Complete Genome	lineage 2	This study
Hangzhou/CO8382/22	PQ505904	2022	Hangzhou, China	Complete Genome	lineage 2	This study
Hangzhou/CO9839/22	PQ505897	2022	Hangzhou, China	Complete Genome	lineage 2	This study
Hangzhou/N124/22	PQ505900	2021	Hangzhou, China	Complete Genome	lineage 1	This study
Hangzhou/N593/21	PQ505899	2021	Hangzhou, China	Complete Genome	lineage 1	This study
Hangzhou/N856/21	PQ505901	2021	Hangzhou, China	Complete Genome	lineage 1	This study
Hangzhou/N1025/21	PQ505903	2021	Hangzhou, China	Complete Genome	lineage 1	This study
Hangzhou/N1376/21	PQ505902	2021	Hangzhou, China	Complete Genome	lineage 1	This study
Hangzhou/N834/22	PQ505896	2022	Hangzhou, China	Complete Genome	lineage 2	This study
Hangzhou/N931/22	PQ505894	2022	Hangzhou, China	Complete Genome	lineage 2	This study
Hangzhou/N1159/22	PQ505895	2022	Hangzhou, China	Complete Genome	lineage 2	This study
OC43	AY391777	1960	United Kingdom	Complete Genome	A	Genbank
VR-759	NC006213	1960	Bethesda, USA	Complete Genome	A	Genbank
Paris	AY585229	2001	Paris, France	Complete Genome	A	Genbank
2145A/2010	KF923888	2010	Shandong, China	Complete Genome	B	Genbank
Belgium 2003	AY903459	2003	Leuven, Belgium	Complete Genome	B	Genbank
3647/2006	KF923900	2006	Beijing, China	Complete Genome	C	Genbank
HK04-01	JN129834	2004	Hong Kong, China	Complete Genome	C	Genbank
HK04-02	JN129835	2004	Hong Kong, China	Complete Genome	D	Genbank

Belgium 2004	AY903460	2004	Leuven, Belgium	Complete Genome	D	Genbank
1783A/10	KP198611	2010	Beijing, China	Complete Genome	E	Genbank
2058A/10	KP198610	2010	Beijing, China	Complete Genome	E	Genbank
MY-U868/12	KX538973	2012	Malaysia: Kuala Lumpur	Complete Genome	F	Genbank
MY-U732/12	KX538971	2012	Malaysia: Kuala Lumpur	Complete Genome	F	Genbank
MY-U464/12	KX538968	2012	Malaysia: Kuala Lumpur	Complete Genome	F	Genbank
12689/2012	KF923902	2012	Beijing, China	Complete Genome	G	Genbank
12694/2012	KF923904	2012	Beijing, China	Complete Genome	G	Genbank
MDS2	MK303620	2014	Lille, France	Complete Genome	G	Genbank
BJ-221	MG197713	2015	Beijing, China	Complete Genome	G	Genbank
YC-207	MG197722	2015	Beijing, China	Complete Genome	G	Genbank
WZ-303	MG197716	2015	Beijing, China	Complete Genome	G	Genbank
China/12/2018	MW532109	2018	Guangdong, China	Complete Genome	H	Genbank
YC-67	MG197719	2018	Beijing, China	Complete Genome	H	Genbank
USA/SC2269/2016	KY684759	2016	Seattle, USA	Complete Genome	I	Genbank
USA/SC831/2016	KY369905	2016	Seattle, USA	Complete Genome	I	Genbank
China/06/2017	MW532116	2017	Guangdong, China	Complete Genome	I	Genbank
China/05/2017	MW532115	2017	Guangdong, China	Complete Genome	I	Genbank
USA/TCNP-00212/2017	MF374985	2017	New Mexico, USA	Complete Genome	I	Genbank
USA/SC9741/2016	KY369907	2016	Seattle, USA	Complete Genome	I	Genbank
HZ-459	MG197723	2016	Beijing, China	Complete Genome	I	Genbank
USA/SC622/2016	KY369906	2016	Seattle, USA	Complete Genome	I	Genbank
China/19/2019	OK318939	2019	Guangzhou, China	Complete Genome	J	Genbank
China/34/2019	OK318944	2019	Yangjiang, China	Complete Genome	J	Genbank
China/21/2019	OK318940	2018	Guangzhou, China	Complete Genome	J	Genbank

China/27/2019	OK318943	2019	Guangdong, China	Complete Genome	J	Genbank
China/24/2019	OK318942	2018	Guangzhou, China	Complete Genome	J	Genbank
Fukushima/H148/2018	LC654454	2018	Fukushima, Japan	Complete Genome	K	Genbank
Fukushima/H478/2018	LC654452	2018	Fukushima, Japan	Complete Genome	K	Genbank
Fukushima/H509/2019	LC654451	2019	Fukushima, Japan	Complete Genome	K	Genbank
China/09/2017	MW532118	2017	Guangdong, China	Complete Genome	K	Genbank
BIME414-37/2019	ON554091	2019	Beijing, China	Complete Genome	K	Genbank
BIME374-10/2019	ON554075	2019	Beijing, China	Complete Genome	K	Genbank
USA/SC0810/2019	MN306041	2019	Seattle, USA	Complete Genome	K	Genbank

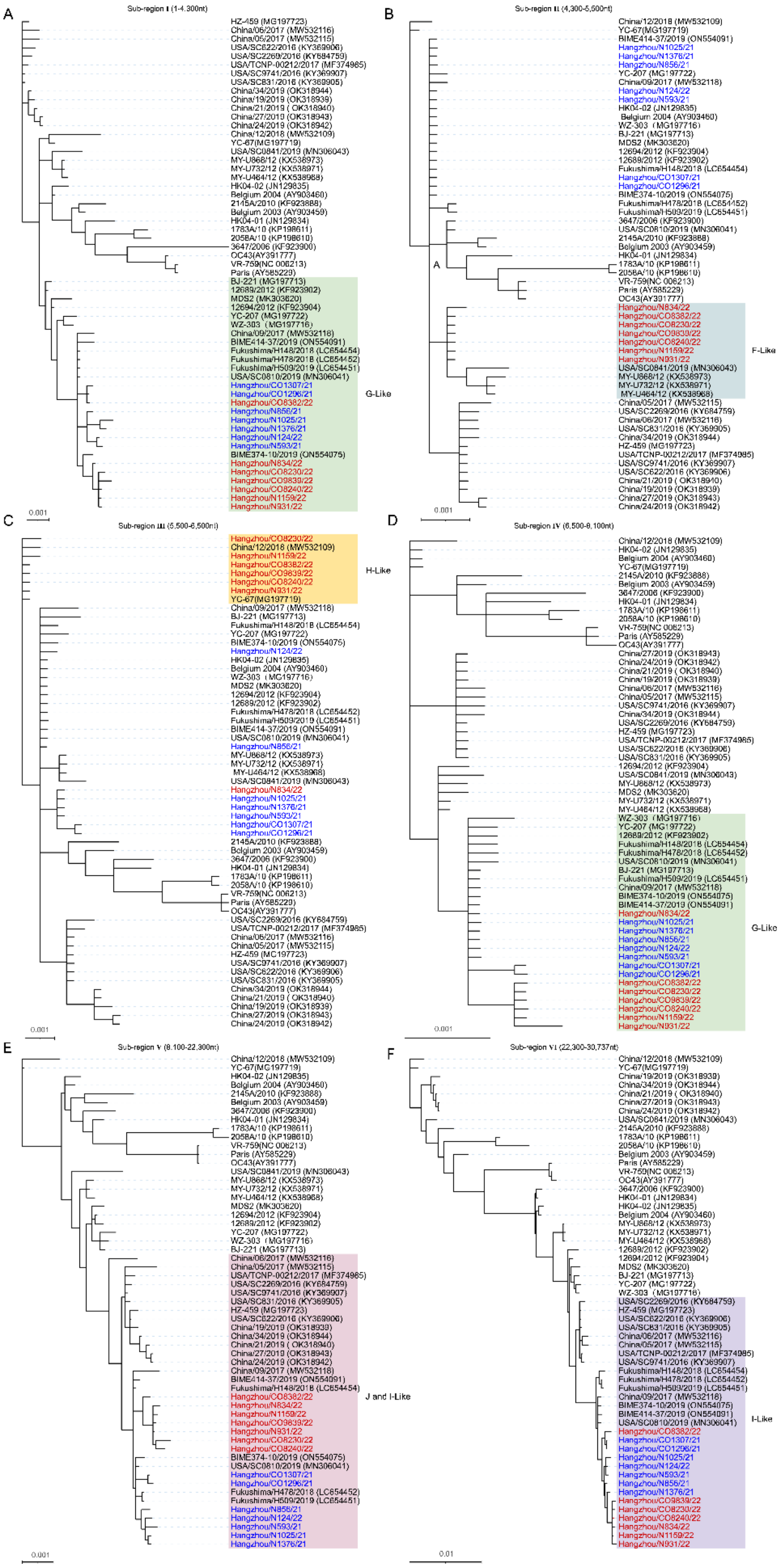

Figure S1. Putative parental genotype determination in sub-genomic regions (sub-regions Ⅰ-Ⅵ) of genotypes K, lineage 1 and lineage 2 recombinants. Breakpoints determined by site information conducted by Bootscan analysis. Green, blue, yellow, pink and purple shades indicate genotypes G, F, H, J and I, and I putative parental genotypes, respectively. Numbering of nucleotide (nt) positions is based on prototype ATCC VR759 reference strain. Taxa names highlighted in blue bold are lineage 1, while those in red bold are lineage 2. The abbreviation of "CO" denotes a co-infected strain, while

“N” indicates a non-co-infected strain. The tree scale bar of an individual tree indicates the substitutions per site.
